# Incidence and risk factors of venous and arterial thromboembolism events in lung cancer patients treated with immune checkpoint inhibitors

**DOI:** 10.3389/fphar.2026.1608497

**Published:** 2026-05-20

**Authors:** Guihua Jin, Ling Tian, Qingqing Jiang

**Affiliations:** 1 Department of Geriatric Oncology, Chongqing University Cancer Hospital, Chongqing, China; 2 Chongqing University Cancer Hospital, Chongqing, China; 3 Chongqing Key Laboratory for the Mechanism and Intervention of Cancer Metastasis, Chongqing University Cancer Hospital, Chongqing, China

**Keywords:** immune checkpoint inhibitors (ICIs), incidence, lung cancer, risk factors, thromboembolism

## Abstract

**Background:**

Thromboembolism (TE), which comprises venous thromboembolism (VTE) and arterial thromboembolism (ATE), represents a significant cause of mortality in patients with lung cancer. It is also associated with considerable morbidity and increased healthcare expenditure. Previous studies have indicated that systemic therapies, including surgery, chemotherapy, radiotherapy, anti-angiogenic therapy, and immunotherapy, can elevate the risk of VTE in patients with lung cancer. However, the risks associated with immune checkpoint inhibitors (ICIs) remain inadequately understood, particularly concerning ATE. This single-center retrospective study aims to assess the incidence of VTE/ATE in a cohort of lung cancer patients undergoing treatment with ICIs, while concurrently identifying potential clinical risk factors.

**Methods:**

A single-center retrospective analysis was conducted of 504 lung cancer patients who received ICIs from January 2019 to December 2022. We examined clinical predictors of TE among all patients. Accordingly, we have defined a predefined critical risk window—from the administration of first ICI dose to 4 weeks after the final dose—established to specifically capture treatment-related thromboembolic events, including VTE and ATE. Follow-up data are obtained through electronic medical records and telephone communication as well as inpatient and outpatient records. Cumulative incidences and risk factors of TE were assessed within a competing-risk framework.

**Results:**

During a mean follow-up period of 27.2 months, TE occurred in 104 out of 504 individuals (20.6%) while on immunotherapy. Among those with TE, 90.4% developed VTE (n = 94), 3.9% experienced ATE (n = 4), and 5.8% had both VTE and ATE (n = 6). The cumulative incidence of TE events at 3, 6, 12 and 24 months were 10.1%,15.7%,19.7% and 20.6%, respectively. For VTE events specifically, the cumulative incidence at these same time points were 9.1%,14.1%,17.5% and 18.7%, respectively. Additionally, the cumulative incidence of ATE events at 3, 6 and 12 months were 1.0%,1.6% and 2.0%, respectively. According to multivariate analysis, four variables were identified as independently associated with TE: age-adjusted Charlson comorbidity index (ACCI) > 8(HR 1.6, 95% CI 1.1–2.3, p = 0.027), the presence of a central venous catheter (CVC) (HR 2.2, 95% CI 1.2–3.9, p = 0.013), D-dimer >0.5 mg/L (HR 1.5, 95% CI 1.0–2.3, p = 0.042), and brain metastases (HR 2.0, 95% CI 1.3–3.1, p = 0.003).

**Conclusion:**

In this study, immune checkpoint inhibitors (ICIs) are associated with a high incidence of VTE in patients with lung cancer, as well as a notable incidence of ATE. In particular, 5.8% of patients with TE experienced both VTE and ATE. This study corroborates the identified risk factors for TE in lung cancer patients undergoing immunotherapy. Targeted thromboprophylaxis may alleviate the burden of TE in this population.

## Introduction

Thromboembolism (TE), which consist of venous thromboembolism (VTE) and arterial thromboembolism (ATE), significantly contributes to morbidity, mortality, and a worse overall prognosis in patients with lung cancer (Sørens et al., 2000; Khorana, 2010; Roopkumar et al., 2021). VTE encompasses deep vein thrombosis (DVT), pulmonary embolism (PE),visceral vein thrombosis (VVT),and catheter-related thrombosis. ATE includes acute coronary syndrome (ACS), acute peripheral arterial occlusion, and stroke. Compared to patients without cancer, the incidence of VTE in cancer patients is reported to be four to nine times higher (Blom et al., 2005; Mulder et al., 2021; Khorana et al., 2007),while the incidence of ATE is approximately two times higher (Navi et al., 2017). Lung cancer, characterized by high incidence and mortality rates, is one of the cancers most commonly associated with TE, with reported incidence rates ranging from 14% to 30% (Bjørnhart et al., 2021; Bagchi et al., 2021; Deschênes-Simard et al., 2021; Nichetti et al., 2019; Roopkumar et al., 2018).

Previous studies have demonstrated that systemic therapies, such as surgery, chemotherapy, radiotherapy, and anti-angiogenic therapy,and immunotherapy, can increase the risk of TE in patients with lung cancer (Mulder et al., 2021; Farge et al., 2019; Madison et al., 2021). Immune checkpoint inhibitors (ICIs), which target programmed cell death receptor-1 (PD-1), programmed cell death ligand-1 (PD-L1), or cytotoxic T-lymphocyte-associated protein 4 (CTLA-4), have become a standard treatment for lung cancer and have significantly improved clinical outcomes for these patients in recent years. Recent small, retrospective cohort studies have reported TE event rates ranging from 8.2% to 24.1% among patients receiving ICIs (Ando et al., 2020; Moik et al., 2021; Khorana et al., 2023). However, despite the increasing use of ICIs, the incidence and high-risk factors of venous thromboembolism (VTE) and arterial thromboembolism (ATE) events associated with ICIs in lung cancer remain under-investigated.

Therefore, the objective of this retrospective cohort study was to evaluate the incidence of VTE and ATE in lung cancer patients undergoing ICIs, as well as to investigate potential clinical risk factors for associated with TE.

## Methods

### Study design and patients

We conducted a retrospective cohort study involving lung cancer patients receiving immune checkpoint Inhibitors (ICIs) therapy at Chongqing University Cancer Hospital from January 2019 to December 2022. All patients in our study cohort received initial combination therapy consisting of ICIs and platinum-based chemotherapy. After completing two to six cycles of combination treatment or upon achieving maximum response, patients who showed no evidence of disease progression were transitioned to ICIs maintenance monotherapy, in accordance with standard clinical practice. In some cases, immunotherapy may have been temporarily suspended and subsequently resumed based on clinical judgment. We defined a critical risk window—from the administration of first ICI dose to 4 weeks after the final dose—to specifically capture treatment-related thromboembolic events, including VTE and ATE. The inclusion criteria were as follows: patients aged ≥18 years, those diagnosed with lung cancer via histopathology, and individuals who received a minimum of two cycles of ICIs.The exclusion criteria included: juvenile patients (<18 years old), individuals with a history of VTE and ATE, and those who declined or were unable to participate in the study. TE, encompassing both VTE and ATE, occurring after the initiation of ICIs therapy were identified through manual chart reviews of electronic medical records. This process involved reviewing radiographic reports including CT (Computed Tomography) scans of the head, chest, abdomen; MRI scans of the head and abdomen; as well as vascular ultrasound of the upper limbs, lower limbs,and neck. VTE was defined to include deep vein thrombosis (DVT), pulmonary embolism (PE),visceral vein thrombosis (VVT),and catheter-related thrombosis. ATE was defined as acute coronary syndrome (ACS), acute peripheral arterial occlusion, and stroke. A history of VTE and ATE referred to occurrences prior to the initiation of ICIs therapy. The detailed protocol of all study-related procedures and analyses was approved by the Ethics Committee at Chongqing University Cancer Hospital (number: CZLS2025044-A). The study adhered strictly to the principles outlined in the Declaration of Helsinki.

### Statistical analyses

All statistical analyses were conducted using R software (version 4.3.2). Patient demographics and clinical characteristics were summarized using descriptive statistics. Count data were compared using the Wilcoxon rank-sum test, as they were not normally distributed; categorical data were compared using the χ2 test. Median follow-up times and survival analysis were calculated via the Log-Rank test. Thrombotic endpoints were examined within a competing-risk framework, treating all-cause mortality as a competing outcome event. Cumulative incidences were calculated using the cmprsk package (version 2.2-10) with the Fine-Gray test applied to subgroups. The cumulative incidences of TE, VTE, and ATE were calculated separately at 3, 6, 12, and 24 months for TE and VTE, and at 3, 6, and 12 months for ATE. Factors associated with TE or VTE or ATE (p < 0.10) in univariate Fine-Gray tests underwent further examination through multivariate analysis utilizing the subdistribution hazard model. p < 0.05 was considered statistically signifificant.

## Results

### Study population characteristics

The study population consisted of 504 individuals with a mean age of 62 years (range: 30–84 years) (Table 1). No patient had undergone surgery within 3 months prior to the initiation of ICIs therapy or during the treatment period, and no additional surgical interventions for other medical conditions were performed during this time. The majority of participants were male (n = 420, 83.3%), diagnosed with non-small cell lung cancer (NSCLC) (n = 460, 91.3%), had stage IV disease (n = 423, 84.5%),and presented with brain metastases at treatment initiation (n = 112, 22.2%). The median follow-up time from ICIs therapy initiation was recorded at approximately 27.2months. The most prevalent histological types of lung cancer included adenocarcinoma (n = 249, 49.4%) and squamous cell carcinoma (n = 187, 37.1%). Camrelizumab emerged as the most frequently utilized agent among patients followed by sintilimab, tislelizumab, pembrolizumab, durvalumab, atezolizumab, nivolumab and toripalimab. Notably, 47.0% (n = 237) of patients received only two to five cycles of ICIs, while 61.3% (n = 309) were treated with first-line of ICIs. Most patients exhibited good Eastern Cooperative Oncology Group (ECOG) performance status (ECOG≤1: 98.6%, n = 497) and favorable age-adjusted Charlson comorbidity index (ACCI) (ACCI≤ 8: 62.1%, n = 313). Only 5.8% (n = 29) of all patients received central venous catheter (CVC).

**TABLE 1:** Clinical characteristics of lung cancer patients with or without TE.

Characteristics	Total (%)	No TE (%)	TE (%)	p Value
Subjects, n	504 (100)	400 (79.4)	104 (20.6)	​
Sex, n (%)	​	​	​	0.844
Male	420 (83.3)	334 (83.5)	86 (82.7)	​
Female	84 (16.7)	66 (16.5)	18 (17.3)	​
Age, (years)	​	​	​	0.202
<65	299 (59.3)	243 (60.8)	56 (53.8)	​
≥65	205 (40.7)	157 (39.2)	48 (46.2)	​
BMI, (kg/m^2^)	​	​	​	0.394
<24	331 (65.7)	257 (64.2)	74 (71.2)	​
24–27.9	140 (27.8)	115 (28.7)	25 (24.0)	​
≥28	33 (6.5)	28 (7.0)	5 (4.8)	​
ECOG, n (%)	​	​	​	0.431
0	20 (4.0)	18 (4.5)	2 (1.9)	​
1	477 (94.6)	377 (94.2)	100 (96.2)	​
2	7 (1.4)	5 (1.2)	2 (1.9)	​
Stage, n (%)	​	​	​	0.031
I-III	78 (15.5)	69 (17.2)	9 (8.7)	​
IV	423 (84.5)	331 (82.8)	95 (91.3)	​
Histological type, n (%)	​	​	​	0.035
Adenocarcinoma	249 (49.4)	186 (46.5)	63 (60.6)	​
Squamous cell carcinoma	187 (37.1)	161 (40.2)	26 (25.0)	​
Other NSCLC	24 (4.8)	19 (4.8)	5 (4.8)	​
SCLC	44 (8.7)	34 (8.5)	10 (9.6)	​
ACCI, n (%)	​	​	​	0.009
≤8	313 (62.1)	260 (65.0)	53 (51.0)	​
>8	191 (37.9)	140 (35.0)	51 (49.0)	​
Caprini score at treatment start, n (%)	​	​	​	0.159
1–2	13 (2.6)	9 (2.2)	4 (3.8)	​
3–4	440 (87.3)	355 (88.8)	85 (81.7)	​
≥5	51 (10.1)	36 (9.0)	15 (14.4)	​
Central venous catheter, n (%)	29 (5.8)	15 (3.8)	14 (13.5)	<0.001
Liver metastases at treatment start, n (%)	60 (11.9)	44 (11.0)	16 (15.4)	0.219
Brain metastases at treatment start, n (%)	112 (22.2)	73 (18.2)	39 (37.5)	<0.001
Bone metastases at treatment start, n (%)	172 (34.1)	130 (32.5)	42 (40.4)	0.131
Prior chemotherapy, n (%)	189 (37.5)	141 (35.2)	48 (46.2)	0.041
Prior radiotherapy, n (%)	75 (14.9)	57 (14.2)	18 (17.3)	0.435
Prior removal of primary tumour, n (%)	62 (12.3)	44 (11.0)	18 (17.3)	0.081
Platelet count (≥350 × 10^9^/L), n (%)	74 (14.7)	55 (13.8)	19 (18.3)	0.246
Hemoglobin (<100 g/L), n (%)	39 (7.7)	29 (7.2)	10 (9.6)	0.421
White blood cell count (>11 × 10^9^/L), n (%)	42 (8.3)	32 (8.0)	10 (9.6)	0.595
D-dimer (>0.5 mg/L), n (%)	266 (52.8)	199 (49.8)	67 (64.4)	0.008
NLR, n (%)	​	​	​	0.084
≤3	202 (40.1)	168 (42.0)	34 (32.7)	​
>3	302 (59.9)	232 (58.0)	70 (67.3)	​
PLR, n (%)	​	​	​	0.111
≤180	258 (51.2)	212 (53.0)	46 (44.2)	​
>180	246 (48.8)	188 (47.0)	58 (55.8)	​
Serum albumin, (g/L)	​	​	​	0.607
<35	115 (22.8)	95 (23.8)	20 (19.2)	​
35–40	189 (37.5)	149 (37.2)	40 (38.5)	​
>40	200 (39.7)	156 (39.0)	44 (42.3)	​
ICIs treatment line, n (%)	​	​	​	0.048
1st-line	309 (61.3)	254 (63.5)	55 (52.9)	​
≥2nd-line	195 (38.7)	146 (36.5)	49 (47.1)	​
ICIs type(s), n (%)	​	​	​	0.169
Pembrolizumab	32 (6.3)	21 (5.2)	11 (10.6)	​
Sintilimab	97 (19.2)	80 (20.0)	17 (16.3)	​
Camrelizumab	240 (47.6)	185 (46.2)	55 (52.9)	​
Tislelizumab	86 (17.1)	74 (18.5)	12 (11.5)	​
Toripalimab	4 (0.8)	4 (1.0)	0	​
Nivolumab	5 (1.0)	4 (1.0)	1 (1.0)	​
Atezolizumab	16 (3.2)	12 (3.0)	4 (3.8)	​
Durvalumab	24 (4.8)	20 (5.0)	4 (3.8)	​
Cycles of ICIs, n (%)	​	​	​	0.818
2–5	237 (47.0)	190 (47.5)	47 (45.2)	​
6–10	152 (30.2)	118 (29.5)	34 (32.7)	​
≥11	115 (22.8)	92 (23.0)	23 (22.1)	​

TE, thromboembolism; BMI, body mass index; ECOG, eastern cooperation oncology group performance status; NSCLC, non-small cell lung cancer; SCLC, small cell lung cancer; ACCI, age-adjusted charlson comorbidity index; NLR, neutrophil to lymphocyte ratio; PLR, platelet to lymphocyte ratio; FDP, fibrin degradation product; ICIs, immune checkpoint inhibitors.

### TE events

TE events occurred in 104 out of 504 individuals (20.6%). Among those with TE, 90.4% developed VTE(n = 94), 3.9% developed ATE (n = 4), 5.8% developed both VTE and ATE (n = 6) (Table 2). Specifically, among the VTE events, DVT accounted for 72 cases (76.6%), catheter-related thrombosis comprised 7 cases (7.5%), DVT with PE represented 6 cases (6.4%), DVT with catheter-related thrombosis included 3 cases (3.2%),PE occurred in 2 cases (2.1%), and VVT was noted in 2 cases (2.1%). Additionally, PE combined with catheter-related thrombosis was observed in 1 case (1.1%), while DVT accompanied by VVT also accounted for 1 case (1.1%). Among the ATE events, stroke accounted for 3 cases (75.0%), while ACS comprised 1 case (25.0%). In the context of combined VTE and ATE events, DVT with stroke represented 3 cases (50.0%), DVT with ACS included 2 cases (33.3%), and DVT with both ACS and Stroke consisted of 1 case (16.7%).

**TABLE 2:** Types of TE events.

Site(s) of TE	N (%)
VTE	94 (90.4)
DVT	72 (76.6)
PE	2 (2.1)
VVT	2 (2.1)
Catheter-related thrombosis	7 (7.5)
DVT + PE	6 (6.4)
DVT + VVT	1 (1.1)
DVT + Catheter-related thrombosis	3 (3.2)
PE + Catheter-related thrombosis	1 (1.1)
ATE	4 (3.9)
ACS	1 (25.0)
Stroke	3 (75.0)
VTE + ATE	6 (5.8)
DVT + ACS	2 (33.3)
DVT + Stroke	3 (50.0)
DVT + ACS + Stroke	1 (16.7)

TE, thromboembolism; VTE, venous thromboembolism; DVT, deep vein thrombosis; PE, pulmonary embolism; VVT, visceral vein thrombosis; ATE, arterial thromboembolism; ACS, acute coronary syndrome.

The majority of VTE events (n = 73, 77.7%) and all ATE events (n = 10, 100%) occurred within the first 12 months following the initiation of ICIs.The cumulative incidence rates of TE at 3, 6, 12, and 24 months were 10.1%, 15.7%, 19.7%, and 20.6%, respectively. For VTE, the corresponding rates were 9.1%, 14.1%, 17.5%, and 18.7%. Regarding ATE, the incidence rates at 3, 6, and 12 months were 1.0%, 1.6%, and 2.0%, respectively (time-to-event analysis is shown in Table 3). Further stratified analysis revealed significant differences in the cumulative incidence of TE events by histological type (p = 0.035),age-adjusted Charlson comorbidity index (ACCI>8) (p = 0.008),Stage IV(p = 0.031),the presence of a CVC(p < 0.001),D-dimer>0.5 mg/L (p = 0.008),brain metastases at treatment start (p < 0.001),and prior chemotherapy (p = 0.049) (Table 4). No signifificant differences were observed in TE rates between various ICIs regimens or based on the number of ICIs cycles. For VTE, significant differences were noted for ACCI>8 (p = 0.037), the presence of a CVC(p < 0.001),D-dimer>0.5 mg/L (p = 0.006), brain metastases at treatment start (p < 0.001), and bone metastases at treatment start (p < 0.001) (Table 5). In terms of ATE, significant differences were found for D-dimer>0.5 mg/L (p < 0.001),liver metastases at treatment star (p < 0.001)t, brain metastases at treatment start (p < 0.001), and number of ICIs cycles (p < 0.001) (Table 6). Due to the retrospective nature of this study, we did not systematically differentiate between symptomatic and asymptomatic events, as symptoms in patients with incidental thrombi may only become apparent upon direct questioning.

**TABLE 3:** Cumulative incidence of TE events.

Site(s) of TE	Cumulative incidence,%			
	3 months	6 months	12 months	24 months
TE	10.1	15.7	19.7	20.6
VTE	9.1	14.1	17.5	18.7
ATE	1.0	1.6	2.0	2.0

TE, thromboembolism; VTE, venous thromboembolism; ATE, arterial thromboembolism.

**TABLE 4:** Cumulative incidence of TE events in different subgroups.

Characteristics	Cumulative incidence,%				
	3 months	6 months	12 months	24 months	p Value
Histological type					
Adenocarcinoma	12.9	20.5	24.5	25.5	​
Squamous cell carcinoma	6.4	10.2	12.8	14.1	0.035
Other NSCLC	8.3	12.2	20.8	20.8	​
SCLC	11.4	13.6	20.5	23.1	​
ACCI					
≤8	8.0	12.1	15.7	17.1	​
>8	13.6	21.5	26.2	26.9	0.008
Stage					
I-III	3.9	9.0	10.3	12.1	0.031
IV	11.3	16.9	21.4	22.5	​
Central venous catheter					
No	9.1	14.5	18.1	19.1	<0.001
Yes	27.6	34.5	44.8	48.7	​
D-dimer					
≤0.5 mg/L	6.3	12.6	15.1	15.7	0.008
>0.5 mg/L	13.5	18.4	23.7	25.4	​
Brain metastases at treatment start					
No	7.9	12.8	16.1	16.7	​
Yes	17.9	25.9	32.1	35.2	<0.001
Prior chemotherapy					
No	9.5	13.7	17.5	17.9	0.049
Yes	11.1	19.1	23.3	25.7	​

TE, thromboembolism; NSCLC, non-small cell lung cancer; SCLC, small cell lung cancer; ACCI, age-adjusted charlson comorbidity index.

**TABLE 5:** Cumulative incidence of VTE in different subgroups.

Characteristics	Cumulative incidence,%				
	3 months	6 months	12 months	24 months	p Value
ACCI					
≤8	7.7	11.5	14.4	15.8	​
>8	11.5	18.3	22.5	23.3	0.037
Central venous catheter					
No	8.0	12.8	16.0	17.0	<0.001
Yes	27.6	34.5	41.4	45.5	​
D-dimer					
≤0.5 mg/L	5.5	11.3	13.0	13.6	0.006
>0.5 mg/L	12.4	16.5	21.5	23.1	​
Brain metastases at treatment start					
No	7.1	11.5	14.3	14.9	​
Yes	16.1	23.2	28.6	31.7	<0.001
Bone metastases at treatment start					
No	7.5	13.0	16.0	17.1	​
Yes	12.2	16.3	20.4	21.7	<0.001
Prior chemotherapy					
No	8.3	12.4	15.6	16.0	​
Yes	10.6	16.9	20.7	23.1	0.059

VTE, venous thromboembolism; ACCI, age-adjusted charlson comorbidity index.

**TABLE 6:** Cumulative incidence of ATE in different subgroups.

Characteristics	Cumulative incidence,%			
	3 months	6 months	12 months	p Value
D-dimer	​	​	​	​
≤0.5 mg/L	0.8	1.3	1.7	<0.001
>0.5 mg/L	1.1	1.9	2.3	​
Liver metastases at treatment start	​	​	​	​
No	0.9	1.4	1.8	​
Yes	1.7	3.3	3.3	<0.001
Bone metastases at treatment start	​	​	​	​
No	0.6	0.9	1.5	​
Yes	1.7	2.9	2.9	<0.001
Cycles of ICIs	​	​	​	<0.001
1–5	0.8	1.7	1.7	​
6–10	1.3	1.3	2.0	​
≥11	0.9	1.7	2.6	​

ATE, arterial thromboembolism; ICIs, immune checkpoint inhibitors.

### Clinical predictors of TE

Univariate competing risks regression analysis revealed that Stage IV(hazard ratio [HR] 2.1, 95% confidence interval [CI] 1.1–4.1, p = 0.036), age-adjusted Charlson comorbidity index (ACCI) > 8 (HR 1.7, 95% CI 1.1–2.5, p = 0.008), the presence of a CVC (HR 3.2, 95% CI 1.8–5.6, p < 0.001),D-dimer >0.5 mg/L (HR 1.7, 95% CI 1.2–2.6, p = 0.008), brain metastases at treatment initiation (HR 2.4, 95% CI 1.6–3.5, p < 0.001), and prior chemotherapy (HR 1.5.95% CI 1.0–2.2, p = 0.048) were associated with an increased risk of developing TE. Conversely, squamous cell carcinoma (HR 0.5, 95% CI 0.3–0.8, p = 0.004) was associated with a decreased risk of TE. Other factors, including other non-small cell lung cancer (NSCLC), small cell lung cancer (SCLC), ICIs treatment line, number of ICIs cycles, prior removal of primary tumour, and neutrophil lymphocyte ratio (NLR), were not significantly associated with TE development. To control for confounding factors, variables exhibiting an association with TE, VTE, or ATE (p < 0.10) in univariate Fine-Gray tests were further analyzed using multivariate subdistribution hazard models. Ultimately, ACCI >8(HR 1.6, 95% CI 1.1–2.3, p = 0.027), the presence of a CVC(HR 2.2, 95% CI 1.2–3.9, p = 0.013), D-dimer >0.5 mg/L (HR 1.5, 95% CI 1.0–2.3, p = 0.042),and brain metastases (HR 2.0, 95% CI 1.3–3.1, p = 0.003) remained statistically signifificant predictors of TE (Table 7).

**TABLE 7:** HRs (95% confifidence intervals) for risk factors associated with TE.

Variables	Univariate		Multivariatea^a^	
	HR(95%CI)	P	HR (95%CI)	P
Histological types				
Adenocarcinoma	1.0	​	1.0	​
Squamous cell carcinoma	0.5 (0.3-0.8)	0.004	0.7 (0.4-1.1)	0.100
Other NSCLC	0.8 (0.3-1.9)	0.580	0.9 (0.4-2.2)	0.820
SCLC	0.9 (0.5-1.7)	0.670	1.0 (0.5-1.8)	0.910
Stage				
I-III	1.0	​	1.0	​
IV	2.1 (1.1-4.1)	0.036	1.3 (0.6-2.6)	0.470
ACCI				
≤8	1.0	​	1.0	​
>8	1.7(1.1-2.5)	0.008	1.6 (1.1-2.3)	0.027
ICIs treatment line				
1st-line	1.0	​	1.0	​
≥2nd-line	1.4 (1.0-2.1)	0.061	1.0 (0.4-2.6)	0.910
Central venous catheter	3.2(1.8-5.6)	<0.001	2.2 (1.2-3.9)	0.013
D-dimer (>0.5 mg/L)	1.7(1.2-2.6)	0.008	1.5(1.0-2.3)	0.042
Brain metastases at treatment start	2.4(1.6-3.5)	<0.001	2.0(1.3-3.1)	0.003
Prior chemotherapy	1.5(1.0-2.2)	0.048	1.2(0.5-3.4)	0.670

TE, thromboembolism; NSCLC, non-small cell lung cancer; SCLC, small cell lung cancer; ACCI, age-adjusted charlson comorbidity index.

^a^ adjusted for histological type, stage, age-adjusted Charlson comorbidity index (ACCI), ICIs, treatment line, central venous catheter, D-dimer, brain metastases at treatment start, cycles of ICIs, prior chemotherapy, prior removal of primary tumour, NLR.

For VTE specifically, univariate analysis indicated that ACCI>8 (HR 1.5, 95% CI 1.0–2.3, p = 0.038), the presence of a CVC (HR 3.4, 95% CI 1.8–6.1, p < 0.001), D-dimer >0.5 mg/L (HR 1.8, 95% CI 1.2–2.8, p = 0.007), and brain metastases at treatment initiation (HR 2.3.95% CI 1.5–3.6, p < 0.001)were associated with an increased risk of VTE. Squamous cell carcinoma (HR 0.6, 95% CI 0.4–0.9, p = 0.013) was associated with a decreased risk of VTE. Multivariate analysis confirmed that the presence of a CVC (HR 2.5, 95% CI 1.3–4.6, p = 0.005), D-dimer >0.5 mg/L (HR 1.6, 95% CI 1.0–2.5, p = 0.040), and brain metastases at treatment initiation (HR 1.9, 95% CI 1.2–3.1, p = 0.006)remained significant predictors of VTE (Table 8). No clinical factors were significantly associated with ATE in univariate analysis.

**TABLE 8:** HRs (95% confifidence intervals) for risk factors associated with VTE.

Variables	Univariate		Multivariatea^a^	
	HR(95%CI)	P	HR (95%CI)	P
Histological types				
Adenocarcinoma	1.0	​	1.0	​
Squamous cell carcinoma	0.6 (0.4-0.9)	0.013	0.7 (0.4-1.2)	0.220
Other NSCLC	0.5 (0.2-1.7)	0.270	0.6 (0.2-2.0)	0.410
SCLC	0.8 (0.4-1.6)	0.500	0.9 (0.4-1.8)	0.740
Stage				
I-III	1.0	0.092	1.0	​
IV	1.8 (0.9-3.5)	​	1.2 (0.6-2.5)	0.630
ACCI				
≤8	1.0	​	1.0	​
>8	1.5 (1.0-2.3)	0.038	1.5 (1.0-2.2)	0.082
ICIs treatment line				
1st-line	1.0	​	1.0	​
≥2nd-line	1.5 (1.0-2.2)	0.068	1.0 (0.4-2.9)	0.990
Central venous catheter	3.4 (1.8-6.1)	<0.001	2.5 (1.3-4.6)	0.005
D-dimer (>0.5 mg/L)	1.8 (1.2-2.8)	0.007	1.6 (1.0-2.5)	0.040
Brain metastases at treatment start	2.3(1.5-3.6)	<0.001	1.9(1.2-3.1)	0.006
Prior chemotherapy	1.5(1.0-2.2)	0.059	1.2(0.4-3.4)	0.760

VTE, venous thromboembolism; NSCLC, non-small cell lung cancer; SCLC, small cell lung cancer; ACCI, age-adjusted charlson comorbidity index.

^a^ Adjusted for histological type, stage, age-adjusted Charlson comorbidity index (ACCI), ICIs, treatment line, central venous catheter, D-dimer, brain metastases at treatment start, cycles of ICIs, prior chemotherapy, prior removal of primary tumour, NLR.

## Discussion

It has long been acknowledged that lung cancer patients have a high incidence of TE, which correlates with poorer prognoses and elevated morbidity levels (Sørens et al., 2000; Khorana, 2010; Nichetti et al., 2019; Okura et al., 2019; Timp et al., 2013). Several studies have reported incidence of VTE in patients with lung cancer receiving platinum-based systemic chemotherapy ranges from 6% to 13% (Madison et al., 2021; Mulder et al., 2019). However, there is currently a lack of data regarding the frequency and risk factors associated with TE in lung cancer patients undergoing treatment with immune checkpoint inhibitors in China. In this retrospective real-world study, we observed a significant occurrence of TE following the initiation of ICIs in lung cancer patients. The cumulative incidence estimates derived from our analysis indicate that nearly one in five patients experiences VTE during ICIs therapy, while approximately one in fifty suffers from ATE. Notably, 5.8% of patients with TE developed both VTE and ATE, suggesting a subset of individuals may have a heightened systemic prothrombotic state. These TE events are clinically significant, particularly given that nearly ninety percent were attributed to VTE.

Several previous studies have suggested a higher cumulative incidence of TE associated with cancer immunotherapy. In a retrospective study involving 1,686 cancer patients treated with ICIs Roopkumar et al. reported cumulative incidence of VTE at 6-month,12-month and 24-month as 7.0%,9.9% and 12.4%, respectively (Roopkumar et al., 2021). A large observational, retrospective pharmacovigilance study demonstrated an increased association of reporting cardiovascular events with ICIs, when compared with other drugs (Roopkumar et al., 2021; Salem et al., 2018). Moik et al. described a cumulative incidence of VTE and ATE in 12.9% and 1.8% of a cohort of 662 patients with a median follow up period of 8.5 months (Moik et al., 2021). In our patient cohort, we observed a higher cumulative incidence of VTE alongside a similar incidence rate for ATE when comparable follow-up periods were examined in the two studies. Given the high mortality risk associated with delayed treatment of ATE, it remains crucial to focus on its management. Therefore, in high-risk patients identified by our model, closer monitoring and proactive consultation with specialists in Stroke Neurology and Cardiology should be considered to support the early detection and management of potential TE events. However, owing to limitations in our data—specifically the lack of granular time-to-event information—this study cannot characterize the temporal distribution of thromboembolic event risk. Consequently, we recommend that prospective studies on this topic prioritize the systematic collection of precise time-to-event data as a data collection objective.

Furthermore, to explore risk factors for TE onset during ICIs administration, we conducted univariate analysis to identify significantly different variables (Table 7). Candidate factors were subsequently identified,and a multivariate analysis was performed. In this study, we report that an age-adjusted Charlson comorbidity index (ACCI) > 8, the presence of a central venous catheter (CVC), D-dimer >0.5 mg/L,and brain metastases are potential risk factors for developing TE.

In 1994, Charlson et al. established a scoring system, the Age-Adjusted Charlson Comorbidity Index (ACCI), to predict the incidence of complications during the perioperative period (Charlson et al., 1994). Patient-related risk factors—including comorbidities such as cardiovascular disease or other conditions (e.g., diabetes, chronic obstructive pulmonary disease, congestive heart failure, renal disease, and liver disease)—contribute to the risk of TE (Falanga et al., 2023). Visual emphysema is an independent risk factor for VTE events and the risk of VTE in chronic obstructive pulmonary disease patients increases with the degree of airway obstruction, invasive mechanical ventilation and chest pain increased the risk of VTE in chronic obstructive pulmonary disease (Dong et al., 2019; Huang et al., 2025). Progressive decreases in renal function increase VTE risk, with the highest risk for fatal pulmonary embolism (PE) in those with an estimated glomerular filtration rate (eGFR) of less than 30 mL/min (Ribic and Crowther, 2016). Data from meta analyses suggest that the annual incidence of VTE in patients with liver disease ranged from 0.3% to 6.3%. The thrombotic risk is particularly elevated in patients with cirrhosis with higher Child-Pugh scores and those with severe liver injury (Ribic and Crowther, 2016; Qi et al., 2015; Rodriguez-Castro et al., 2012; Lisman et al., 2013). However, due to the retrospective nature of our study and the structure of our dataset, we are unable to provide a robust, statistically reliable analysis of individual comorbidity. The number of events (particularly for ATE) within our cohort is limited. Subdividing the cohort further by multiple individual comorbidities would result in cell sizes too small for meaningful multivariate analysis, increasing the risk of unstable estimates. Although the use of the ACCI has certain value in exploring the overall burden of comorbidities, it does not enable us to distinguish the specific effects of different diseases on the risks of VTE and ATE. In future prospective studies, systematic collection of specific types and severity of comorbidities in the ACCI should be considered, in order to better understand their individual roles.

Recently, Kahl A et al. investigated the prognostic value of ACCI in ovarian cancer patients (Kahl et al., 2017) and demonstrated that a high ACCI was associated with shorter overall survival (OS). Furthermore, ACCI remained a significant prognostic factor for OS in multivariate analysis. Similarly, another study involving 1,476 gastric cancer patients who underwent radical gastrectomy showed that the 5-year OS rate in low ACCI group was significantly higher than that in high ACCI group; multivariate analysis indicated that ACCI was an independent risk factor for OS (Lin et al., 2019). Although previous studies have reported on the impact of ACCI on long-term prognosis in patients with other malignant tumors (such as pancreatic and colorectal cancer) (Dias-Santos et al., 2015; Wu et al., 2015), but its predictive value of risk factors for developing TE with immunotherapy in lung cancer has not been previously addressed. In the present study, we identified a positive correlation between an age-adjusted Charlson comorbidity index (ACCI) > 8 and TE. Thus, ACCI may serve as a potential risk factor for TE in patients with lung cancer which receiving immunotherapy.

D- Dimer, a soluble fibrin degradation product generated through plasmin-mediated cleavage of cross-linked fibrin, serves as a key biomarker of coagulation activation and secondary fibrinolysis. It is routinely employed to exclude TE and to assess the risk of VTE recurrence, as well as to guide decisions on the optimal duration of anticoagulant therapy. Several studies have evaluated D-dimer levels’ predictive value regarding TE among cancer patients (Koch et al., 2023; Di et al., 2023; Ay et al., 2009). Di W et al. found that the serum D-dimer levels were significantly elevated in the VTE group compared to those without VTE (Di et al., 2023). Another investigation involving 526 cancer patients from the University Hospital Heidelberg (Germany) revealed that D-dimer exhibited the highest positive predictive value of 96% at concentrations of 9.9 mg/L and a negative predictive value of 100% at a level of 0.6 mg/L for identifying VTE cases (Koch et al., 2023). However, D-dimer levels naturally increase with age, which complicates their interpretation, particularly in elderly cancer patients. Therefore, the use of a fixed 0.5 mg/L cutoff as a predictive threshold for TE risk in our study is subject to certain limitations. To address this issue, multiple studies have proposed age-adjusted D-dimer thresholds (Righini et al., 2001; Fatah et al., 2025; Righini et al., 2014; Robert-Ebadi et al., 2021; Stals et al., 2022). Currently, the most widely adopted approach involves multiplying the patient’s age by 10 (ug/L) for individuals over 50 years old to improve specificity (Righini et al., 2014). The use of a uniform D-dimer cutoff in our study was dictated by standardized institutional laboratory references, which do not account for age-related physiological changes. Consequently, this approach may have compromised diagnostic specificity, particularly among older individuals, and could have led to an overestimation of thromboembolic risk in this subgroup.

Individuals diagnosed with cancer frequently require long-term central venous access for the administration of anti-cancer therapies, parenteral nutrition, or transfusion of blood products and components. However, a study conducted by Ashrani AA et al. Identified that central venous catheter (CVC) is an independent risk factor associated with an 8.5-fold increase in the likelihood of VTE among cancer patients (Ashrani et al., 2016). A prospective observational study evaluating the safety of CVC in patients with cancer reported a 4.5% incidence rate of CVC-related thrombosis (Kim et al., 2010). Our findings further indicate a positive correlation between the central venous catheter (CVC) and TE in patients in cancer patients undergoing treatment with ICIs, which aligns with previous research outcomes.

Brain metastases occur in 20%-40% of patients with cancer,and their frequency has increased over time (Di Lorenzo and Ahluwalia, 2017). Brain metastases represent a group of neoplasms that constitute the most common type of brain tumour in adults. Lung cancer is the predominant source of these brain metastases. Up to 20% patients with brain metastases present with VTE (Lopez-Ruz et al., 2021). VTE is a well-recognized complication associated with metastatic brain tumors (Riedl and Ay, 2019). However, the association between brain metastases and VTE related to immunotherapy has not been documented in the literature. In our study,we found that among patients with brain metastases, the incidence of TE was nearly twice as high compared to those without brain metastases. Therefore, further research is warranted to elucidate the correlation between brain metastasis and the occurrence of VTE in lung cancer patients.

The present study has several limitations. First, our data are derived from a single health system-based retrospective cohort with a relatively small sample size. However, the combination of this limited sample size and detailed patient follow-up facilitated by the use of a unified electronic medical record system, along with manual chart review, allowed us to effectively capture VTE and ATE events. Another limitation of the current study is the potential omission of asymptomatic TE events due to this follow-up methodology. As it was a retrospective study, certain variables-such as anticoagulation status and PD-1/PD-L1 expression-were not recorded, which might influence our results. Furthermore, anticoagulation at treatment initiation could serve as an indicator of underlying comorbidities that were not accounted in this cohort study. Future investigations should aim to include prospective homogenous cohorts to mitigate any bias related to these issues. Additionally, patients who received alternative treatments—such as chemotherapy alone, targeted therapy alone, immunotherapy alone, or other combined anti-tumor treatment methods—were not included as controls in this study. The risk of thrombosis during the immune-maintenance phase was not analyzed further. The absence of concurrent, randomly assigned comparisons across different treatment regimens restricts our capacity to directly assess relative risk differences among various therapeutic modalities. Although we captured the overall ACCI score, inconsistent coding granularity for specific comorbidities (e.g., severity of liver/renal disease or diabetes with end-organ damage) in our retrospective records precluded reliable, standardized recategorization. Future well-designed prospective cohort studies or subgroup analyses of randomized controlled trials are therefore warranted to enable direct comparisons of thrombosis risks across distinct anti-tumor treatment strategies.

In conclusion, patients undergoing immunotherapy for lung cancer are at an increased risk of TE. An age-adjusted Charlson comorbidity index (ACCI) > 8, the presence of a central venous catheter (CVC), D-dimer >0.5 mg/L,and brain metastases were identified as risk factors for the development of TE. Future research should involve multicenter, large-scale cohorts with enhanced statistical power to validate the findings of this study. Such efforts will aid in identifying effective predictors and implementing early interventions aimed at improving patients’ quality of life and prolonging their survival.

## Data Availability

The original contributions presented in the study are included in the article/supplementary material, further inquiries can be directed to the corresponding author.
